# A Novel Spectrum Contrast Mapping Method for Functional Magnetic Resonance Imaging Data Analysis

**DOI:** 10.3389/fnhum.2021.739668

**Published:** 2021-09-08

**Authors:** Qin Yu, Zenglin Cai, Cunhua Li, Yulong Xiong, Yang Yang, Shuang He, Haitong Tang, Bo Zhang, Shouyun Du, Hongjie Yan, Chunqi Chang, Nizhuan Wang

**Affiliations:** ^1^Artificial Intelligence and Neuro-Informatics Engineering (ARINE) Laboratory, School of Computer Engineering, Jiangsu Ocean University, Lianyungang, China; ^2^Department of Neurology, The Affiliated Suzhou Science and Technology Town Hospital of Nanjing Medical University, Suzhou, China; ^3^Center for Brain Science and Learning Difficulties, Institute of Psychology, Chinese Academy of Sciences, Beijing, China; ^4^Department of Radiology, Affiliated Lianyungang Hospital of Xuzhou Medical University, Lianyungang, China; ^5^Department of Neurology, Guanyun People’s Hospital, Guanyun, China; ^6^Department of Neurology, Affiliated Lianyungang Hospital of Xuzhou Medical University, Lianyungang, China; ^7^Health Science Center, School of Biomedical Engineering, Shenzhen University, Shenzhen, China; ^8^Pengcheng Laboratory, Shenzhen, China

**Keywords:** functional magnetic resonance imaging, spectrum contrast mapping, fast fourier transform, resting-state, task-state, test-retest, Parkinson’s disease

## Abstract

Many studies reported that spontaneous fluctuation of the blood oxygen level-dependent signal exists in multiple frequency components and changes over time. By assuming a reliable energy contrast between low- and high-frequency bands for each voxel, we developed a novel spectrum contrast mapping (SCM) method to decode brain activity at the voxel-wise level and further validated it in designed experiments. SCM consists of the following steps: first, the time course of each given voxel is subjected to fast Fourier transformation; the corresponding spectrum is divided into low- and high-frequency bands by given reference frequency points; then, the spectral energy ratio of the low- to high-frequency bands is calculated for each given voxel. Finally, the activity decoding map is formed by the aforementioned energy contrast values of each voxel. Our experimental results demonstrate that the SCM (1) was able to characterize the energy contrast of task-related brain regions; (2) could decode brain activity at rest, as validated by the eyes-closed and eyes-open resting-state experiments; (3) was verified with test-retest validation, indicating excellent reliability with most coefficients > 0.9 across the test sessions; and (4) could locate the aberrant energy contrast regions which might reveal the brain pathology of brain diseases, such as Parkinson’s disease. In summary, we demonstrated that the reliable energy contrast feature was a useful biomarker in characterizing brain states, and the corresponding SCM showed excellent brain activity-decoding performance at the individual and group levels, implying its potentially broad application in neuroscience, neuroimaging, and brain diseases.

## Introduction

### Low-Frequency Fluctuations in Human Brain Activity

Low-frequency fluctuation is an intrinsic property of human brain activity. Biswal et al. (1995) used functional magnetic resonance imaging (fMRI) technology to initially demonstrate that spontaneous low-frequency fluctuation was synchronized in the bilateral motor cortices of the brain at rest. Inspired by this study, low-frequency fluctuations in the sensorimotor and auditory cortices were also shown to have a high degree of temporal correlation (Biswal et al., 1997; Cordes et al., 2001); this phenomenon was also observed in many other brain areas such as the visual network, default mode network, and others (Lowe et al., 1998; Raichle et al., 2001; Greicius et al., 2003; Kiviniemi et al., 2004). Several studies reported that low-frequency fluctuation observed in fMRI signals was highly connected with spontaneous neuronal activities (Logothetis et al., 2001; Goldman et al., 2002). There is also growing research into low-frequency fluctuations, and a well-known example provided the theoretical basis for the spontaneous fluctuation referred to as resting-state networks (Damoiseaux et al., 2006; De Luca et al., 2006; Robinson et al., 2009; Zuo et al., 2010). Resting-state fMRI (rs-fMRI) connectivity has also been applied to reveal abnormalities in intrinsic connectivity of the salience network in patients with psychiatric disorders (Dutta et al., 2014). For example, Yang et al. (2016) found that the amplitude of low-frequency fluctuations and functional connectivity based on resting-state data exhibited consistent alterations in the bilateral anterior insula of subjects with major depressive disorder. Another notable example is the method of amplitude of low-frequency fluctuation (ALFF) (Zang et al., 2007), and study has demonstrated that ALFF can be used as a metric of brain diseases (Han et al., 2011) and for decoding brain activity (Yang et al., 2018). Furthermore, low-frequency fluctuation has been extensively applied in research into mental illness (Cui et al., 2020), neurological disease (Dutta et al., 2014), cognition (Fransson, 2005; Welsh et al., 2010), and neuroplasticity (Lewis et al., 2009; Di et al., 2012; Wu et al., 2020).

### High-Frequency Fluctuations in Human Brain Activity

Two decades ago, there was a relatively small body of literature regarding high-frequency fluctuations (>0.08 Hz) in brain activity (Devrim et al., 1999). Animal research showed that the cat’s primary visual cortex exhibited frequency fluctuations (<0.3 Hz) (Steriade et al., 1993). Several studies were performed that primary auditory cortex regions responded to acoustic stimuli in the frequency domain of 0.1–0.5 Hz (Filippov and Frolov, 2004; Filippov et al., 2007, 2008). In terms of high-frequency fluctuations in human brain activity, Devrim et al. (1999) reported that the sensory threshold of the human visual system might be regulated by slow cortical potentials. Moreover, Salvador et al. (2008) used a measure of functional connectivity describing to show that the high- (0.17–0.25 Hz) and middle- (0.08–0.17 Hz) frequency intervals are prominent in several limbic and temporal regions. In recent years, there has been an increasing number of publications on high-frequency fluctuation (0.08–1 Hz) and progression of low-frequency fluctuation (0.01–0.08 Hz) research. For example, Gohel et al. (2018) found differences in the regions of the visual cortex and dorsal attention in the frequency band of 0.027–0.25 Hz between healthy subjects and patients with psychosis. Jiang et al. (2019) showed that the strength of dynamic functional connectivity was weakened in the thalamus subregion of patients with schizophrenia in the frequency domain of 0.073–0.198 Hz. In an examination of frequency fluctuation, Jiang et al. (2019) demonstrated new-onset, drug-naive (unmedicated) increased functional connectivity in the rolandic network in the frequency band (0.027–0.198 Hz). Furthermore, Yaesoubi et al. (2017) found that structured spectro-temporal variability existed in resting-state connectivity and can reveal differences and similarities between clinical and healthy populations. Functional connections in the high-frequency band gradually attracted research attention, and several studies reported that the high-frequency band had a similar network connection in the low-frequency fluctuation (Boubela et al., 2013; Smith-Collins et al., 2015; Trapp et al., 2018).

### What Do We Propose?

The aforementioned studies indicated that this signal exists in multiple frequency components and fluctuates over time. However, they largely focused on low- or high-frequency information of a given time course separately, which ignored the relationship between the two frequencies. Thus, in this paper, we first assumed that the energy contrast between the low- and high-frequency band for each voxel (in terms of the overall frequency spectrum band) is relatively stable for a certain brain state. We proposed a novel spectrum contrast mapping (SCM) method to validate the assumption of energy contrast based on the designed experiments.

The remainder of this paper is organized as follows: the theory and methods related to SCM will be presented first, followed by the experimental designs. We also describe the initial tests using SCM on task-related, resting state, test-retest datasets and further apply SCM to Parkinson’s disease (PD) resting-state dataset. Finally, the results will be presented together with interpretations and conclusions related to the advantages and limitations of our proposed model.

## Theory and Methods

### SCM Framework

**FIGURE 1:**
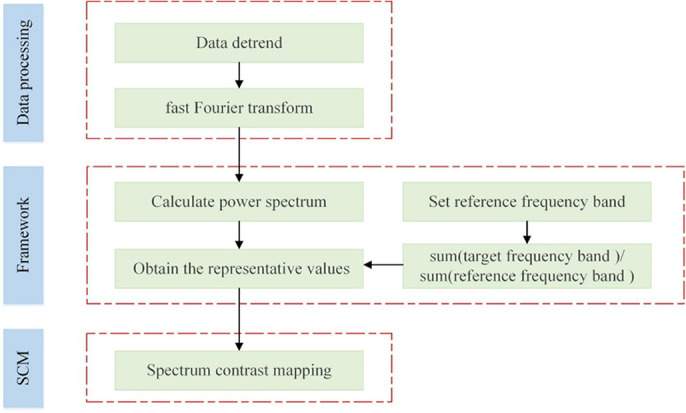
The framework of the proposed SCM method.

The proposed SCM framework was displayed in Figure 1. According to Figure 1, the experimental fMRI signals were first preprocessed by temporal detrending, and then we performed fast Fourier transformations to obtain the corresponding power spectra of brain voxels. Segmentation of the spectral signal yielded the low-frequency band (0.01–0.1 Hz) and high-frequency band (0.1–0.25 Hz), and the latter was used as the reference benchmark. Two representative values were, respectively selected from the above two frequency bands. Ultimately, SCM values were obtained by calculating the ratio of two representative values of each brain voxel signal.

### SCM Formulation

Given a signal_*f∈L*^1^ (ℝ)_, where_*A_k_(t)*_is the amplitude function,_ϕ_*k*_(*t*)_ is the phase function, and _*k*_ is the number of components. For the time series_*f(t)*_of an individual element, it is expanded by Fourier series:

(1) $$f\,\left(t\right)=\sum_{k=1}^{K}A_{k}\,\left(t\right)\,e^{i\,2\,\pi \,\phi_{k}\,\left(t\right)}=\sum_{k=1}^{K}\left[a_{k}\,\cos\left(2\,\pi \,f_{k}\,t\right)+b_{k}\,\sin\left(2\,\pi \,f_{k}\,t\right)\right]$$

where_*a_k_*_ and_*b_k_*_ are the amplitudes of the real frequency components, _*f_k_*_ is the frequency band,_*n*_is the number of different frequency bands, value of *k*:1 to *n*, for the following definition with SCM:

(2) $$L\,F\,R\,V=R\,V\,f\in \left[l\,o\,w,m\,i\,d\right]\,\left(\sqrt{a_{k}^{2}\,\left(f_{k}\right)+b_{k}^{2}\,\left(f_{k}\right)}\right),$$

(3) $$H\,F\,R\,V=R\,V\,f\in \left(m\,i\,d,h\,i\,g\,h\right]\,\left(\sqrt{a_{k}^{2}\,\left(f_{k}\right)+b_{k}^{2}\,\left(f_{k}\right)}\right),$$

(4) S⁢C⁢M=L⁢F⁢R⁢V/H⁢F⁢R⁢V,

where RV represents the representative value, LFRV represents the value selected in the target low-frequency band, and HFRV represents the value selected in the high-frequency reference band. The low-, middle-, and high-frequency cut values in this study are set to 0.01, 0.1, and 0.25 Hz, respectively.

### Evaluating Indicator

The corrcoef indicator is introduced to evaluate the effectiveness of the proposed SCM. For given matrices A and B, the correlation coefficient can be calculated as in formula (5)

(5) $$c\,o\,r\,r\,c\,o\,e\,f\,\left(A,B\right)=\frac{c\,o\,v\,\left(v\,e\,c\,\left(A\right),v\,e\,c\,\left(B\right)\right)}{\sqrt{v\,a\,r\,\left(v\,e\,c\,\left(A\right)\right)\,v\,a\,r\,\left(v\,e\,c\,\left(B\right)\right)}},$$

where A and B are two matrices with the same dimensions, respectively; _*cov(•)*_, _*var(•)*_, and_*vec(•)*_represent the calculation operation of covariance, variance, and vector transform, respectively (Wang et al., 2016a).

## Experimental Designs

All the algorithms and toolkits used were implemented in the Windows 10 operating system and configured as follows: Intel(R) Core (TM) i7-7700HQ CPU @ 2.80 GHz and 16GB RAM. They were run on the MATLAB 2019a platform, and all preprocessing steps were performed using dpabi software (Yan et al., 2016). The first ten time points were removed. The remaining volume images were preprocessed sequentially by slice timing, realignment, normalization, and spatial smoothing with an 8 mm full-width half-maximum Gaussian kernel.

### Visual Task-Related Dataset

In the visual task experimental test, six subjects were informed of the purpose of this study before taking part in the visual task-related experiment. The designed visual paradigm was OFF-ON-OFF-ON-OFF-ON in a 40-s block. In the “ON” state, the visual stimulus was a radial blue/yellow checkerboard, reversing at 7 Hz. In the “OFF” state, the participants were required to focus on a cross at the center of the screen. More experimental details can be found elsewhere (Ren et al., 2014; Wang et al., 2017).

### Resting-State Dataset With Eyes Open and Closed

The functional images were obtained using an echo-planar imaging (EPI) sequence with the following parameters: 33 axial slices, thickness/gap = 3.5/0.7 mm, in-plane resolution = 64 × 64, repetition time (TR) = 2,000 ms, echo time (TE) = 30 ms, flip angle 90°, field of view (FOV) = 200 × 200 mm^2^. Each condition consisted of 240 functional volumes. For this experiment, 45 healthy and normal subjects were selected, with eyes closed (EC) twice and once with eyes open (EO). The T1-weighted MP-RAGE image was acquired with the following parameters:128 sagittal slices, slice thickness/gap = 1.33/0 mm, in-plane resolution = 256 × 192, TR = 2,530 ms, TE = 3.39 ms, inversion time (TI) = 1,100 ms, flip angle = 7°, FOV = 256 × 256 mm^2^. The detailed protocol was described by Liu et al. (2013).

### Test-Retest Resting-State Dataset

The test-retest resting-state fMRI dataset of 25 normal participants is available by downloading from https://www.nitrc.org/projects/nyu_trt (Shehzad et al., 2009; Zuo et al., 2010). Each participant was scanned three times at rest with a Siemens Allegra 3.0 Tesla MRI scanner, and the fMRI data for each subject consisted of 197 continuous EPI functional volumes (TR = 2 s, TE = 25 ms, flip angle = 90°, slice number = 39, matrix = 64 × 64, FOV = 192 × 192 mm^2^, voxel size = 3 × 3 × 3 mm^3^). A high-resolution T1-weighted magnetization prepared gradient echo (MPRAGE) sequence was also obtained for each participant, with the following acquisition parameters: TR = 2,500 ms, TE = 4.35 ms, TI = 900 ms, flip angle = 8°, slice number = 176, FOV = 256 × 256 mm^2^.

### Parkinson’s Disease Resting-State Dataset

There were a total of 45 participants involved in this dataset, which had three contrast groups, i.e., cognitively normal PD with severe hyposmia (PD-SH) with 15 subjects, cognitively normal patients with PD with no/mild hyposmia (PD-N/MH) with 15 subjects, and healthy controls (HCs) with 15 subjects. Each participant was scanned at rest with a Siemens Magnetom Verio 3.0 T scanner with a 32-channel head coil. A high-resolution T1-weighted magnetization prepared gradient echo sequence was obtained for each participant. Meanwhile, the rs-fMRI scans (8 min, eyes closed) were also acquired with the following parameters: TR = 2.5 s, TE = 30 ms, slice number = 39, thickness = 3 mm, FOV = 192 × 192 mm^2^, matrix = 64 × 64, flip angle = 80°. The detailed protocol was described by Yoneyama et al. (2018). This dataset with accession number ds000245 was obtained from the OpenfMRI database.^1^

## Results and Analysis

### Visual Task-Related Experiment

**FIGURE 2:**
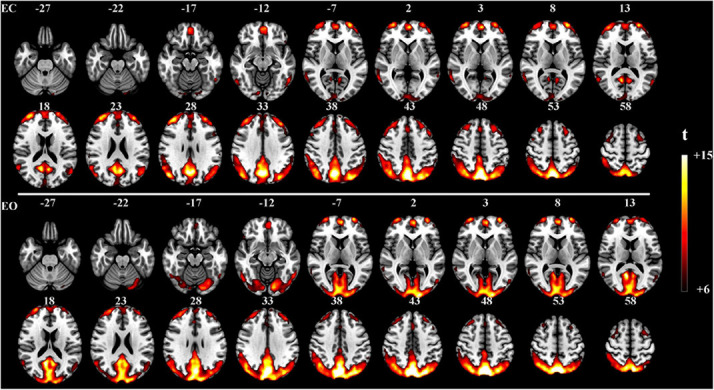
Extracted SCM maps for six subjects in the visual task-related dataset.

The proposed SCM was applied to extract the visual task-related voxel-wise brain activity maps of six subjects and to generate the corresponding SCM maps. The z-scored SCM maps were transformed with Z-score operation and were displayed in Figure 2. Most subjects had strong SCM values in visual task-related areas such as the occipital, fusiform, calcarine, and lingual gyri. Unlike many brain network separation models under the assumption of statistical independence or sparse distribution (Wang et al., 2012, 2013, 2016b, 2015; Yaesoubi et al., 2017; Yao et al., 2013; Shi et al., 2017; Shi and Zeng, 2018), the SCM paid more attention to the spectrum energy contrast of each voxel.

### Resting-State Experiment With Eyes Open and Closed

**FIGURE 3:**
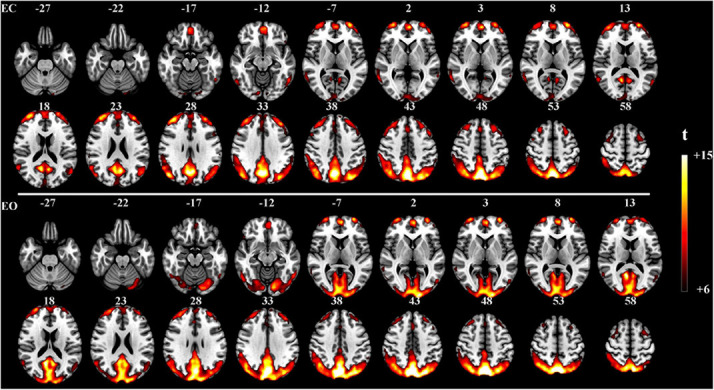
One-sample *t*-test SCM maps under the EC (*n* = 45, *t* > 6.0, *p* < 0.0005, FDR corrected) and EO (*n* = 45, *t* > 6.0, *p* < 0.0005, FDR corrected) resting-state conditions.

**FIGURE 4:**
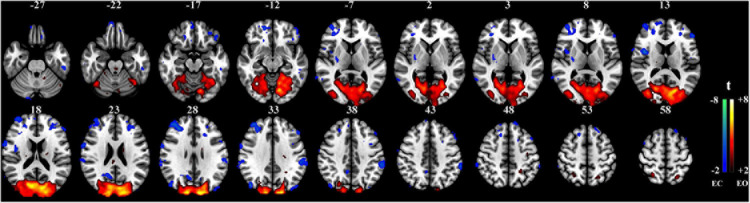
Paired *t*-test SCM maps between the EC and EO resting-state conditions (*n* = 45, *t* value > 2.0, *p* < 0.05, FDR corrected).

The proposed method was used in resting-state experiments for extracting brain networks in the EC and EO conditions among 45 subjects, and the corresponding SCM maps for EC and EO states were generated. The one-sample *t*-test SCM maps with false discovery rate (FDR) correction (Genovese et al., 2002) were calculated and displayed in Figure 3. Notably, SCM highlighted the famous default mode networks involving areas such as the medial prefrontal cortex and posterior cingulate cortex/precuneus for both the EC and EO conditions (Fransson, 2005; Buckner et al., 2008). Furthermore, parts of the dorsolateral prefrontal cortex, occipital cortex, cuneus, calcarine, lingual, fusiform, and cerebellum showed significant enhancement in the EO state compared to the EC state. Paired *t*-tests were performed to quantitatively analyze differences in SCM maps between the EC and EO conditions. The paired *t*-test maps in Figure 4 show that there were significant differences in bilateral visual areas for EC versus EO. The main differences were in the bilateral visual cortices (Brodmann areas 18 and 19) involving the occipital_mid, lingual, occipital_sup, calcarine, and cuneus. There were also differences in the fusiform and cerebellum_6 areas. These results indicated that energy contrast could be a useful biomarker to characterize the degree of activity of brain regions at rest under different conditions.

### Test-Retest Resting-State Experiment

**FIGURE 5:**
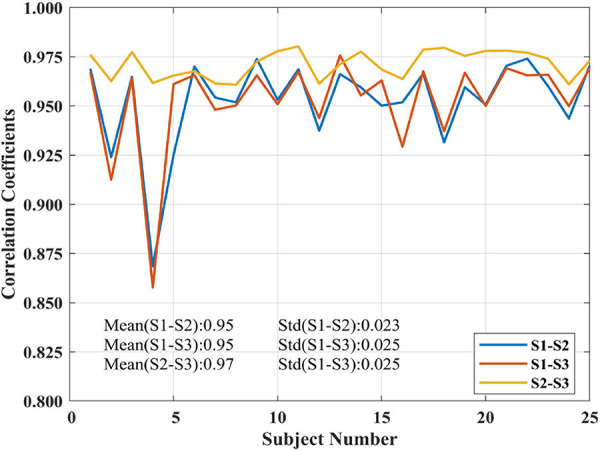
Correlation coefficients of SCM maps among three sessions for 25 subjects in the test-retest resting-state dataset.

The proposed SCM method was subjected to test-retest reproducibility validation in decoding brain activity for three sessions at rest among 25 subjects, and individual SCM maps were generated. The interval between sessions 1 and sessions 2 and 3 were 5–16 months (mean 11 ± 4 months). The intercorrelation results among the SCM maps of each subject derived from different sessions were shown in Figure 5. The SCM maps of a given subject had close correlations among three sessions, especially in the intrasession scan (<1 h apart) of sessions 2 and 3. The marked mean and SD values in Figure 5 clearly showed that the proposed SCM had good individual reproducibility in test-retest resting-state experiments.

**FIGURE 6:**
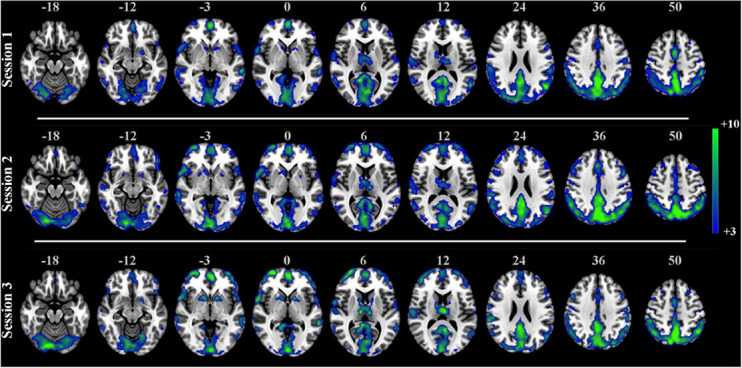
One-sample *t*-test SCM maps separately derived from three sessions in the test-retest resting-state dataset (*n* = 25, *t* > 3.0, *p* < 0.001, FDR corrected).

The one-sample *t*-test SCM maps at the group level for each session were calculated and displayed in Figure 6. The visual areas, default network, and frontal lobe were simultaneously active, and there were no noisy regions in ventricles or large blood vessels. We also calculated the correlation coefficients between group-level SCM maps from three sessions. The intercorrelation coefficients of the group-level SCM maps were 0.90 (sessions 1 and 2), 0.92 (sessions 1 and 3), and 0.95 (sessions 2 and 3), demonstrating high reproducibility. Based on the test-retest validation results, the spectrum contrast feature was an effective biomarker with high reproducibility at both the individual and group levels.

### Resting-State Experiment in Parkinson’s Disease

**FIGURE 7:**
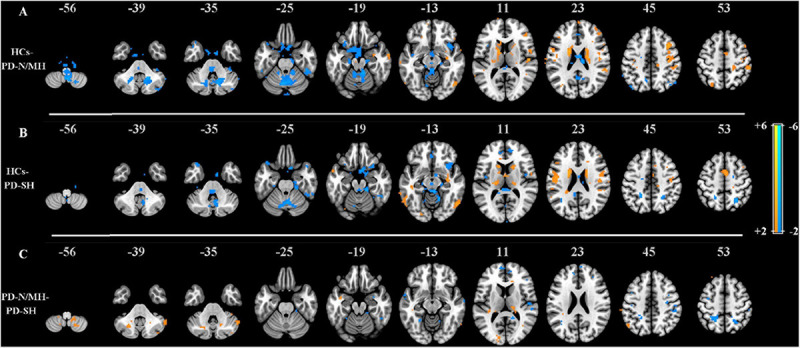
**(A)** Z–statistical difference maps between the HCs (warm color) and PD-N/MH (cool color) (*n* = 15, *z* > 2.0, *p* < 0.05, Tukey-Kramer correction). **(B)** Z–statistical difference maps between the HCs (warm color) and PD-SH (cool color) (*n* = 15, *z* > 2.0, *p* < 0.05, Tukey-Kramer correction). **(C)** Z–statistical difference maps between the PD-N/MH (warm color) and PD-SH (cool color) (*n* = 15, *z* > 2.0, *p* < 0.05, Tukey-Kramer correction).

The proposed SCM method was firstly applied to PD dataset to decode the aberrant neuronal energy contrast of brain activity among three groups of participants, generating the individual SCM maps in each group, i.e., PD-SH, PD-N/MH, and HCs. Further, these corresponding SCM maps were analyzed with analysis of variance (ANOVA) model with a post hoc Tukey-Kramer correction, and the results were shown in Figure 7.

By observing Figures 7A,B, compared to PD-N/MH and PD-SH, the values of SCM in many regions were significantly enhanced in HCs, including the supplementary motor area, frontal cortex, postcentral gyrus, caudate nucleus, rolandic operculum, precentral gyrus, etc. Compared to HCs, PD-N/MH and PD-SH showed extensive differences in regions such as vermis, cerebellum, insula, etc. Notably, PD-SH highlighted regions of the frontal and parietal gyrus compared to HCs (showed in Figure 7B), while PD-N/MH highlighted the regions of the precuneus and superior temporal gyrus compared to HCs (showed in Figure 7A). Further, to investigate the effects of severe hyposmia in Parkinson’s disease, a comparison was made between PD-N/MH and PD-SH, where the results were displayed in Figure 7C. The results showed that compared to PD-N/MH, PD-SH differed in the frontal, parietal and temporal gyrus; in contrast, PD-N/MH showed significant differences in the part regions of the cerebellum and thalamus, etc. These results were well consistent with previous studies (Yoneyama et al., 2018; Han et al., 2019), which demonstrated that the proposed SCM could capture the aberrant neuronal energy contrast between the patients of PD-N/MH and PD-SH compared to HCs.

## Discussion

### Discussion of Experimental Results

The results of the visual task experiment in Figure 2 demonstrated that the proposed SCM could be effective to decode block task-evoked brain activity under the assumption that the energy contrast between low- and high-frequency bands for each voxel is reliable. However, in our task-related experiment, the stimulus task was modulated by the block design. In the future, we will fully explore SCM performance under event-related, block design, and natural stimulus conditions.

With regard to the resting-state experiment under the EC and EO conditions, the results of the two-sample paired *t*-tests in Figure 5 revealed differences in activated regions for the two different states in terms of the energy contrast between the low- and high-frequency bands. Generally speaking, under the EO condition, the low-high frequency energy contrast of the brain activity in several areas (i.e., the occipital, cuneus, calcarine, lingual, part of fusiform, and cerebellum) was significantly enhanced compared to the EC condition. These findings further verified that the low-high frequency energy contrast used in SCM is an effective biomarker for characterizing resting brain activity.

Reproducibility in neuroimaging has gained more and more attention (Zeng et al., 2009; Wang et al., 2016b; Chen et al., 2018; Zuo et al., 2019; Cohen et al., 2021). In this paper, a public test-retest resting-state dataset was used to test the repeatability of the proposed SCM method. As shown in Figure 5, the mean individual reproducibility of the SCM maps from three sessions across 25 subjects was within [0.9528, 0.9713], which showed a high degree of reliability. Figure 6 showed that the group-level SCM maps from three separate sessions were highly correlated with the smallest correlation coefficient equal to 0.90, further demonstrating good reproducibility of the proposed SCM at the group level.

Application of SCM in Parkinson’s disease demonstrated that PD-SH and PD-N/MH compared to HCs extensively differed in several areas (i.e., the vermis, cerebellum, insula, etc.). As shown in Figure 7, the spontaneous neuronal energy contrast captured by SCM can be sensitive, and these aberrant regions can be utilized as an effective biomarker to distinguish diseased individuals from the healthy, which may be related to the pathology of brain diseases. In the future, we will apply the SCM to explore the energy contrast biomarker of more mental illnesses and neurological diseases.

### Discussion of Preprocessing Impact

In recent years, head movements were found that can introduce artifactual differences in spectral power (Kim et al., 2014) and test-retest reproducibility (Yan et al., 2013). Thus, to demonstrate clearly all effects of head motion and global signal regression (GSR) on the proposed SCM, we explored the effects on SCM from the aforementioned two preprocessing operations. The head motion parameters and global signal were regressed out using multiple linear regression analysis in EC-EO and test-retest resting-state datasets, respectively. Specifically, Supplementary Figures 1–4 showed the differences in the head motion regression (without GSR and with GSR) by one-sample test and paired *t*-test. It revealed that head motion regression significantly impacted regions involving part of the frontal, temporal, lingual, and occipital cortex, cerebellum and fusiform, etc. (Kim et al., 2014), especially under the condition of the GSR. However, test-retest reproducibility analysis in Supplementary Figure 5 showed no significant effect of head movement regression (without GSR and with GSR) on reproducibility in SCM (Yan et al., 2013).

### Discussion of Frequency Bands Selection

According to Section 2 Theory and Methods, the proposed SCM method assumes that the energy contrast between the low- and high-frequency bands for each voxel is reliable and credible. Thus, SCM performance mainly depends on the band selection of the reference frequency. In this study, the high-frequency band (0.1–0.25 Hz) was empirically selected as the reference due to the low-frequency oscillation of brain activity measured by fMRI, which was also used in previous studies (Luo et al., 2020; Yang et al., 2020). Theoretically, the proposed SCM highlights the signals with a more concentrated low-frequency property and higher energy contrast value, which can form different functional regions, representing biometric features of brain activity from BOLD signal.

Additionally, stable energy distribution in sub-bands of the frequency domain has been reported by Yuen et al. (2019), which implies that the SCM may be used to locate these areas by setting different target and reference frequency bands. Specifically, Supplementary Figure 6 showed results of the energy contrast between two intrinsic mode functions frequency bands (Yuen et al., 2019) (target bands: 0.01–0.05 Hz, 0.05–0.1 Hz) and the high-frequency bands (reference benchmark bands: 0.1–0.25 Hz). It provided clear evidence that reliable energy contrast existed between the intrinsic mode functions frequency bands in the form of ratios instead of the amplitude values. Moreover, the proposed SCM can be treated as a new feature extraction method based on quantifying the reliable energy contrast of the voxel’s time course, which can be applied to feature extraction, pattern classification (Savio et al., 2011; Sridhar et al., 2017), etc.

### Discussion of SCM V.s. ALFF, fALFF, and PerAF

With regard to low-frequency fluctuations, Zang et al. (2007) proposed the amplitude of low-frequency fluctuation (ALFF) as a characterization of the spontaneous brain activity at the frequency domain. Zou et al. (2008) reported fractional ALFF (fALFF) for rs-fMRI signal analysis, which can be regarded as a standardized ALFF-like metric at each voxel. Recently, Jia et al. (2020) proposed the percent amplitude of fluctuation (PerAF) for rs-fMRI, focusing on the percent change compared to the mean value for each voxel time series.

Theoretically, the proposed SCM is fundamentally different from the three methods mentioned above, but in terms of metric representation, the proposed SCM is related to fALFF. Indeed, fALFF is an inspiring study with wide application in analyzing rs-fMRI signals, but the SCM method proposed in this paper is theoretically different to fALFF. For example, fALFF concerns the percentage of low-frequency bands over the entire frequency domain. By contrast, SCM mainly considers the reliable energy contrast between the target bands and the reference ones.

To compare the test-retest reliability of SCM and fALFF, the intraclass correlation coefficient (ICC) was calculated between each pair of the three sessions in the test-retest resting-state dataset. As shown in Supplementary Figures 7, 8, we could observe that the reliability of gray matter was significantly higher than the one of white matter; also, the short-term reliability was higher compared to the long-term one in the metrics of fALFF and SCM. Further, the reliability of SCM was significantly higher than the one of fALFF in both the short- and long-term test-retest, especially in the cingulate, occipital cortex, and frontal gyrus.

By comparing the results of the one-sample test between SCM and fALFF, as, respectively, shown in Figure 3 and Supplementary Figure 9, it was demonstrated that the proposed SCM effectively decoded the difference of brain activity in visual areas under the EC-EO rest conditions, consistent with fALFF. Additionally, the main difference was marked by the yellow box in Supplementary Figure 9, including temporal and precentral gyrus. Further, we performed the paired *t*-test to detect the differences in voxels between the fALFF and SCM maps under the EC-EO conditions (showed in Supplementary Figure 10). Under the EC condition, compared to fALFF, SCM significantly enhanced regions, including precuneus cortex, frontal and fusiform gyrus, and rectus, while fALFF highlighted regions of temporal, postcentral, occipital, precentral, frontal gyrus compared to SCM. Under the EO condition, SCM showed extensive differences in areas, such as frontal, temporal, fusiform gyrus, and precuneus cortex, while fALFF highlighted areas, such as temporal, frontal, postcentral, precentral, cingulum, parietal gyrus, caudate nucleus, and putamen. Additionally, numerous regions were located by fALFF in the white matter under both the EC and EO conditions.

The paired *t*-test plots of SCM comparing with both ALFF and perAF for EC and EO conditions were shown in Supplementary Figure 11, respectively. The results showed that the SCM mainly highlighted temporal, occipital, frontal, parietal, gyrus, cuneus, cerebellum, and the precuneus cortex, compared to the perAF under the EC condition. Compared to the SCM under the EC condition, the perAF mainly enhanced part of regions in the frontal, lingual gyrus, cerebellum, and many ventricles. Comparing ALFF with SCM, ALFF particularly highlighted the ventricles and the lingual gyrus, while SCM mostly enhanced the temporal, occipital, frontal, parietal, gyrus, cuneus white matter areas in the EC condition. The difference between SCM and perAF and ALFF in the EO condition was similar to the EC condition, clearly seen in Supplementary Figure 11.

### Discussion of Physiological Explanation of Energy Contrast

Regarding the interaction of signals of different frequency bands, there existed many shreds of evidence in electroencephalogram (EEG) (Lindsley, 1952) studies, and this phenomenon was called cross-frequency coupling (CFC), including three categories: phase-phase coupling, phase-amplitude coupling, amplitude-amplitude coupling. CFC phenomenon can be observed in brain regions, such as the hippocampus, prefrontal, and sensory cortex, and CFC was considered as a potential mechanism for higher cognitive functions such as spatial and temporal memory encoding and integration of perceptual information (Händel and Haarmeier, 2009; Canolty and Knight, 2010; De Hemptinne et al., 2013; Chaieb et al., 2015; Hyafil et al., 2015). Furthermore, Rosso et al. (2001) proposed the relative wavelet energy (RWE) to represent information about the corresponding degree of importance of the different frequency bands present in the EEG. Then, Rosso et al. (2006) showed that the epileptic recruitment rhythm observed during seizure development is well described in terms of the RWE. Furthermore, Rosso (2007) applied RWE to epileptic EEG and found significantly decreasing activity in RWE associated with the frequency band 0.8–3.2 Hz (δ activity) at seizure onset, indicating a predominance of the mid-frequency band 3.2–12.8 Hz (theta and alpha bands) at seizure onset. Besides, in terms of fMRI signal, Wang et al., 2015 used the relative wavelet packet energy (RWPE) criterion to select the threshed wavelet tree nodes and to form the sparse approximation coefficients set of original fMRI signal, which helped to improve the brain functional networks identification. In addition, the fALFF also can be treated as a special energy contrast method for decoding brain activity from fMRI signals. In terms of the proposed SCM, it can be viewed as a general energy contrast based method to explore the changes in energy contrasts between different frequency bands under the assumption that the energy contrast between the frequency bands of voxels is stable during spontaneous brain activity. In the future, the other modalities of vivo brain imaging technologies such as EEG, functional near-infrared spectroscopy (fNIRS) (Naseer and Hong, 2015), magnetoencephalography (MEG) (Hansen et al., 2010), etc., will be used to help reveal the more concrete physiological explanation of energy contrasts in SCM by the concurrent EEG-fMRI, fNIRS-fMRI, or integrated MEG-fMRI.

## Conclusion

We proposed a novel but simple voxel-wise brain activity decoding model (SCM), which assumed reliable energy contrast between low- and high-frequency bands for each voxel. The results of the visual task, rest with eyes open and closed, test-retest resting-state, and Parkinson’s disease experiments confirmed that SCM was good at characterizing the energy contrast and showed excellent reliability in decoding brain activity. Indeed, the energy contrast between low- and high-frequency bands was an effective biomarker in characterizing the brain states at both individual and group levels, and can be utilized to reveal the aberrant regions which might relate to the pathology of brain diseases. The proposed SCM method has potential application for neuroscience, neuroimaging and brain diseases.

## Data Availability Statement

The original contributions presented in the study are included in the article/Supplementary Material, further inquiries can be directed to the corresponding author/s.

## Ethics Statement

The studies involving human participants were reviewed and approved by the IRBs of Beijing Normal University, New York University (NYU) and Nagoya University. The patients/participants provided their written informed consent to participate in this study.

## Author Contributions

QY: conceptualization, methodology, validation, formal analysis, and writing—original draft. ZC: conceptualization, validation, funding acquisition, and writing—review and editing, CL, YX, and YY: software, investigation, and writing—review and editing. SH, HT, BZ, and SD: data curation, investigation, and writing—review and editing. HY: conceptualization, methodology, validation, formal analysis, writing—review and editing, and funding acquisition. CC and NW: conceptualization, resources, writing—review and editing, supervision, funding acquisition, and project administration. All authors contributed to the article and approved the submitted version.

## Conflict of Interest

The authors declare that the research was conducted in the absence of any commercial or financial relationships that could be construed as a potential conflict of interest.

## Publisher’s Note

All claims expressed in this article are solely those of the authors and do not necessarily represent those of their affiliated organizations, or those of the publisher, the editors and the reviewers. Any product that may be evaluated in this article, or claim that may be made by its manufacturer, is not guaranteed or endorsed by the publisher.

## Footnotes

1
https://openfmri.org/
