# Supplementary material for: A Novel Spectrum Contrast Mapping Method for Functional Magnetic Resonance Imaging Data Analysis
Source: Front Hum Neurosci. 2021 Sep 8;15:739668. doi: 10.3389/fnhum.2021.739668 (PMC8455948; doi:10.3389/fnhum.2021.739668)
Supplement: Supplementary file 1 [file Data_Sheet_1.pdf]

Supplementary file for A Novel Spectrum Contrast Mapping Method for Functional Magnetic Resonance Imaging Data Analysis

Qin Yu[1#], Zenglin Cai[2#], Cunhua Li[1], Yulong Xiong[1], Yang Yang[3], Shuang He[1], Haitong Tang[1], Bo Zhang[4], Shouyun Du[5], Hongjie Yan[6*], Chunqi Chang[7,8*], Nizhuan Wang[1*]

Figure S1. (A) One-sample t-test SCM results on the EC-EO resting-state datasets with head motion regression (without GSR) (n = 45, t > 6.0, p < 0.0005, FDR corrected). **(B)** One-sample t-test SCM results on the EC-EO resting-state datasets with head motion regression (with GSR) (n = 45, t > 6.0, p < 0.0005, FDR corrected).

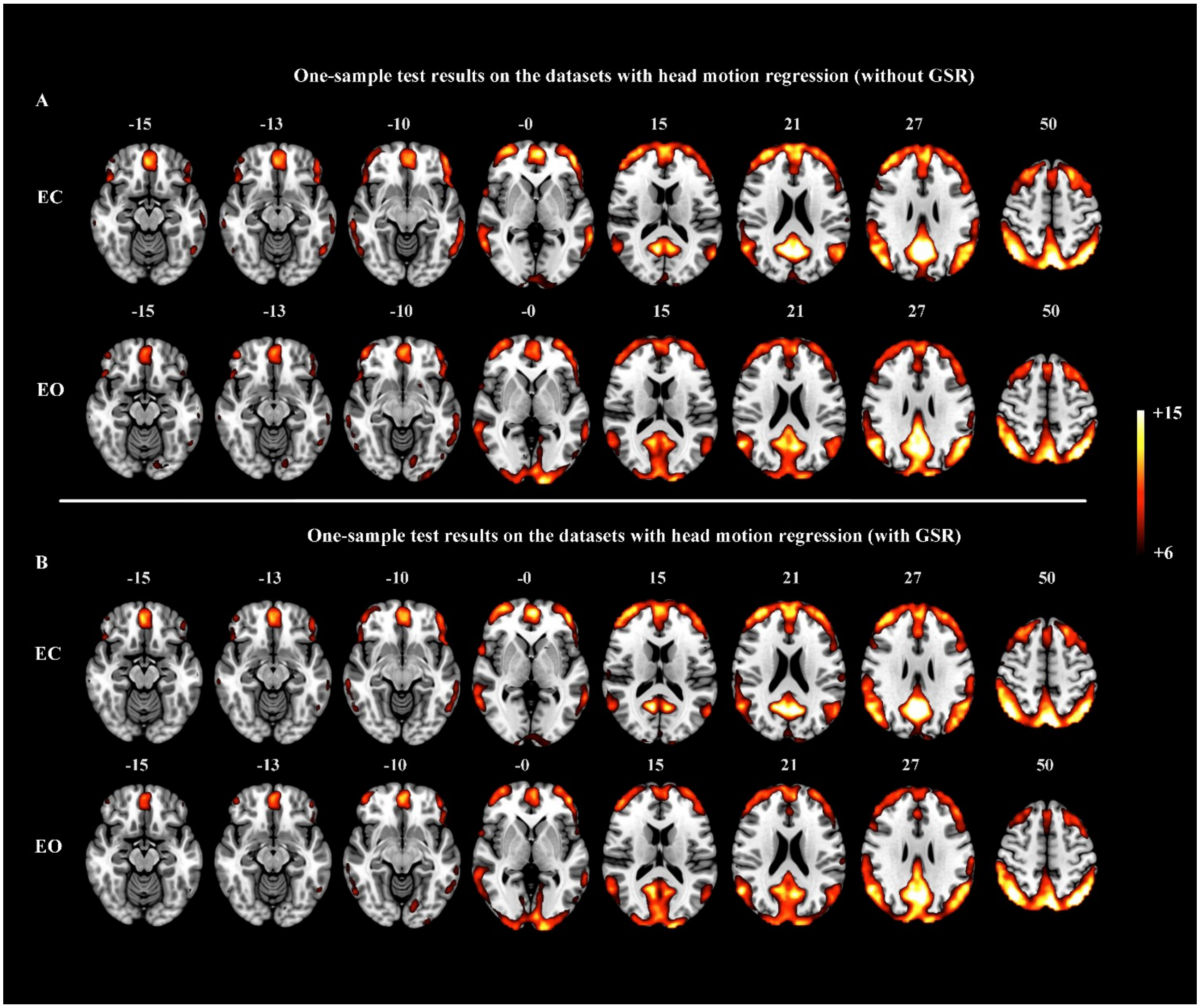

Figure S2. (A) Paired t-test SCM results between EC-EO resting-state datasets without head motion regression (hot color) and with head motion regression (without GSR) (winter color) (n = 45, t value > 6.0, p < 0.005, FDR corrected). **(B)** Paired t-test SCM results between EC-EO resting-state datasets without head motion regression (hot color) and with head motion regression (with GSR) (winter color) (n = 45, t value > 6.0, p < 0.005, FDR corrected).

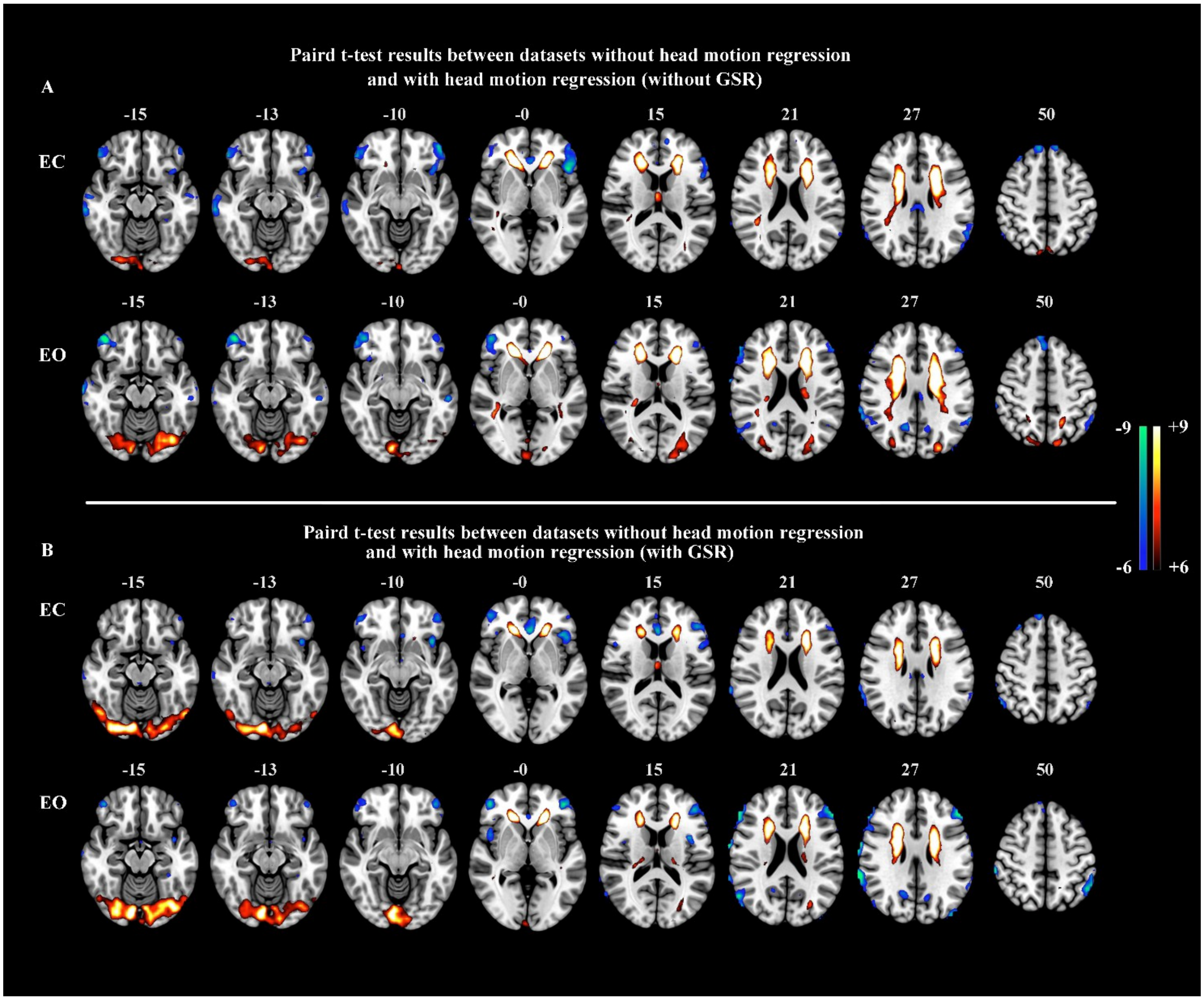

Figure. S3. **(A)** One-sample t-test SCM results on the test-retest datasets with head motion regression (without GSR) (n = 25, t > 3.0, p < 0.001, FDR corrected, three sessions). **(B)** One-sample t-test SCM results on the test-retest resting-state datasets with head motion regression (with GSR) (n = 25, t > 3.0, p < 0.001, FDR corrected, three sessions).

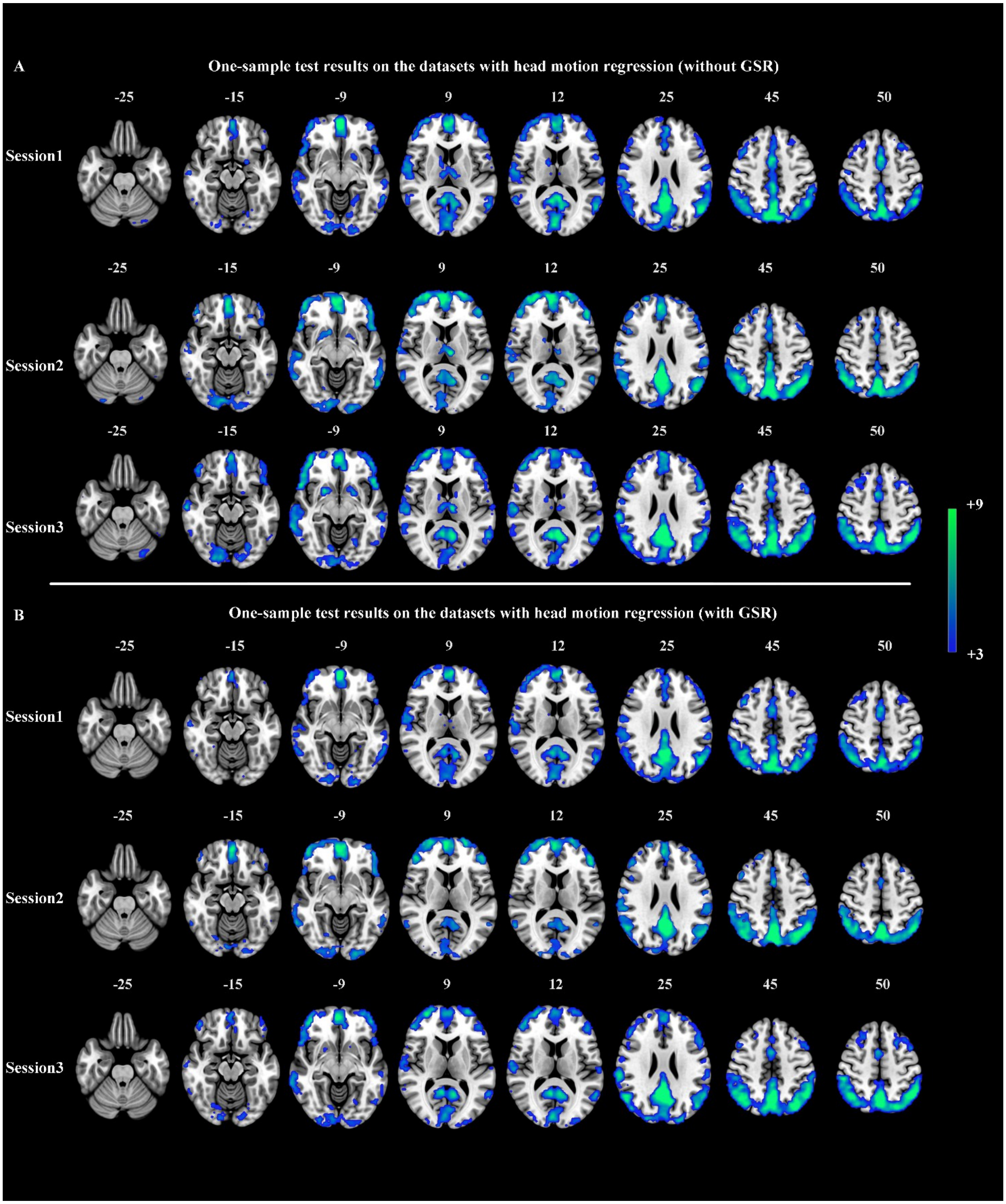

Figure S4. **(A)** Paired t-test SCM results between test-retest resting-state datasets without head motion regression (hot color) and with head motion regression (without GSR) (winter color) (n = 25, t value > 3.0, p < 0.01, FDR corrected, three sessions). **(B)** Paired t-test SCM results between test-retest datasets without head motion regression (hot color) and with head motion regression (with GSR) (winter color) (n = 25, t value > 3.0, p < 0.01, FDR corrected, three sessions).

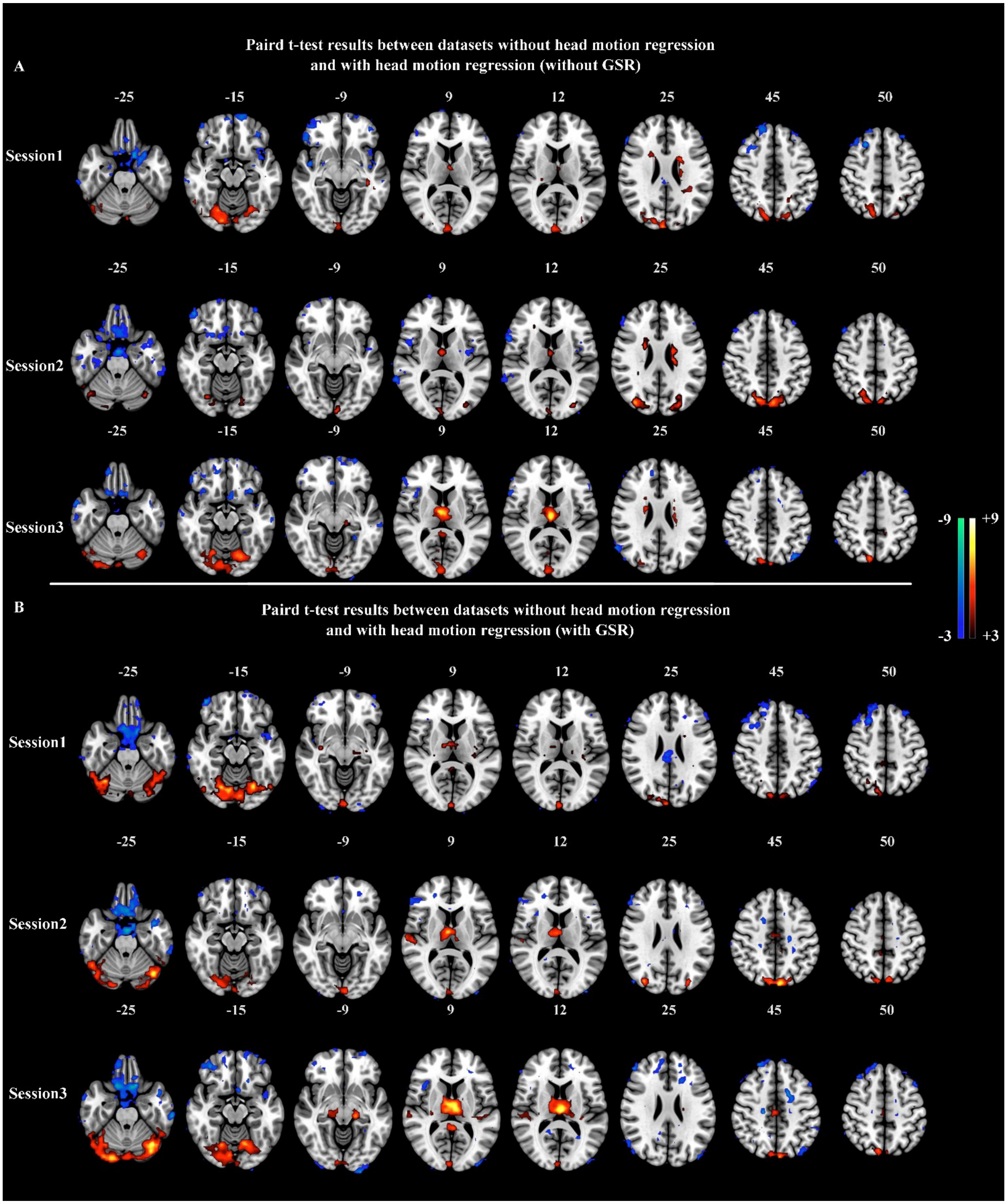

Figure S5. (A) Correlation coefficients of SCM maps among three sessions for 25 subjects in the test-retest resting-state dataset with head motion regression (without GSR). **(B)** Correlation coefficients of SCM maps among three sessions for 25 subjects in the test-retest resting-state dataset with head motion regression (with GSR).

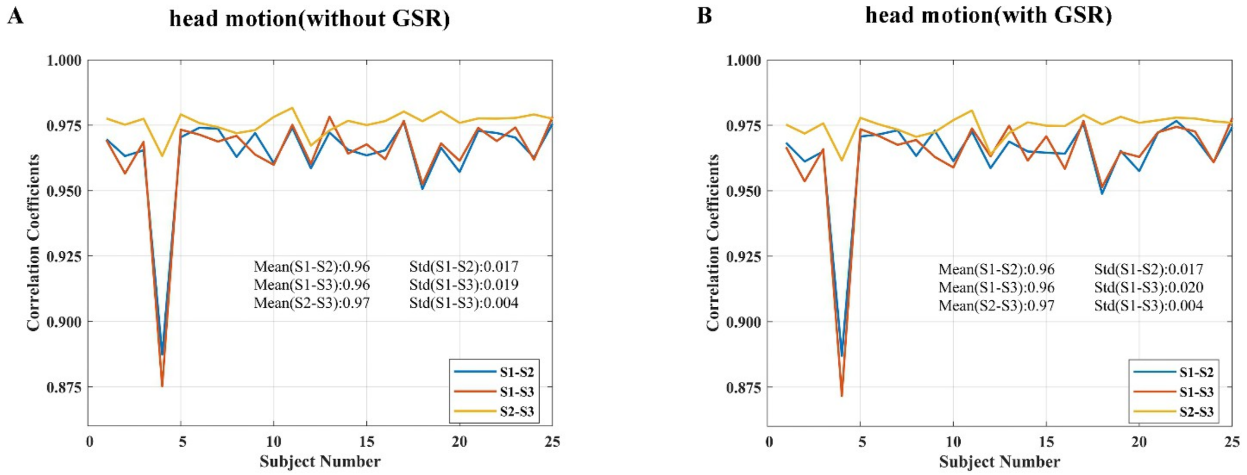

Figure S6. **(A)** One-sample t-test SCM results on the EC-EO resting-state datasets without head motion regression (without GSR) (n = 45, t > 6.0, p < 0.0005, FDR corrected, LF:0.01-0.05Hz, HF:0.1-0.25 Hz). **(B)** One-sample t-test SCM results on the EC-EO resting-state datasets with head motion regression (with GSR) (n = 45, t > 6.0, p < 0.0005, FDR corrected, LF:0. 05-0.1 Hz, HF:0.1-0.25 Hz).

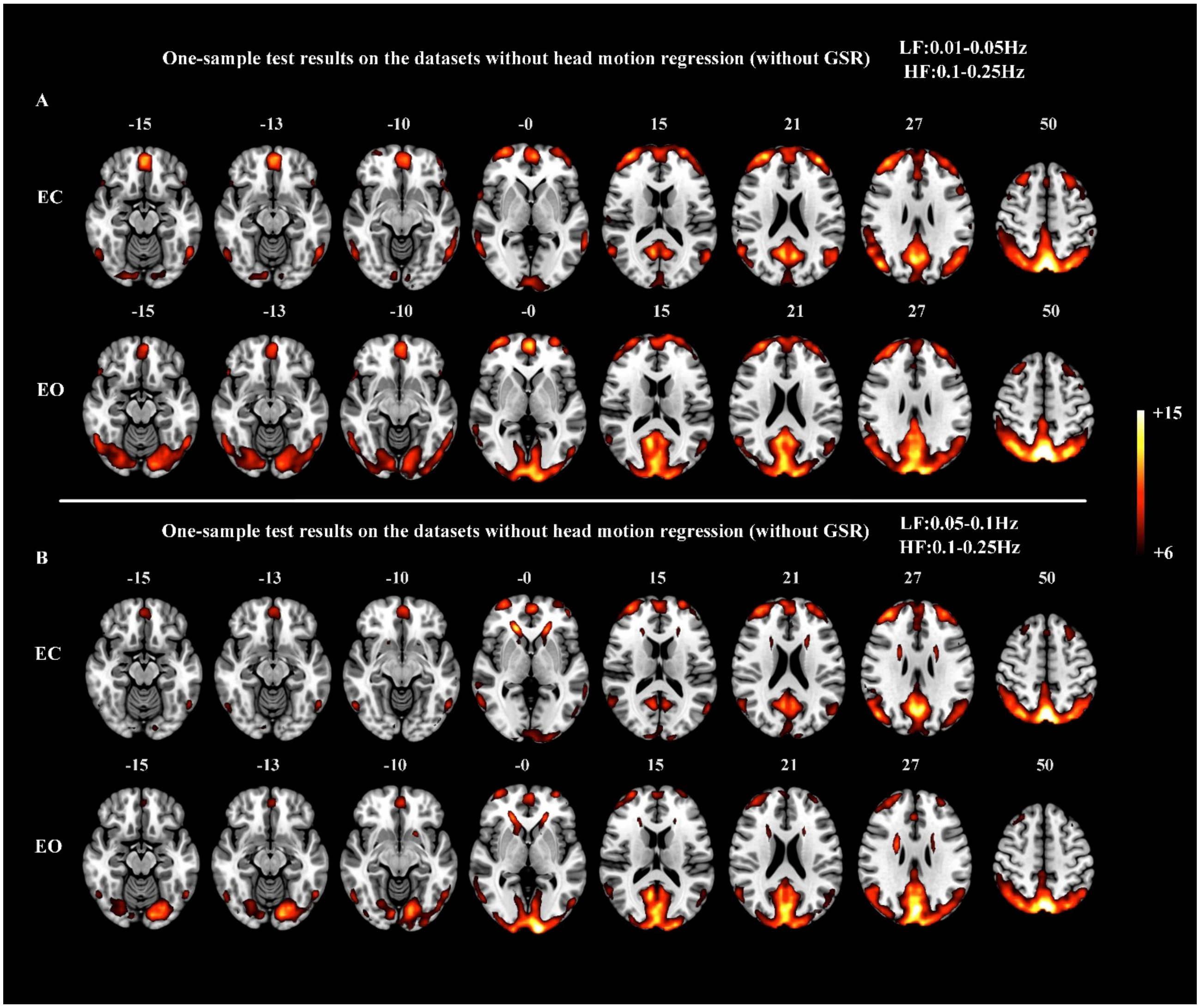

Figure S7. ICC maps for long-term (**A**, session 1 against session 2) and short-term (**B**, session 2 against session 3) reliability between fALFF (left, minus mean and divided by standard deviation) and SCM (right, minus mean and divided by standard deviation) on the test-retest resting-state dataset.

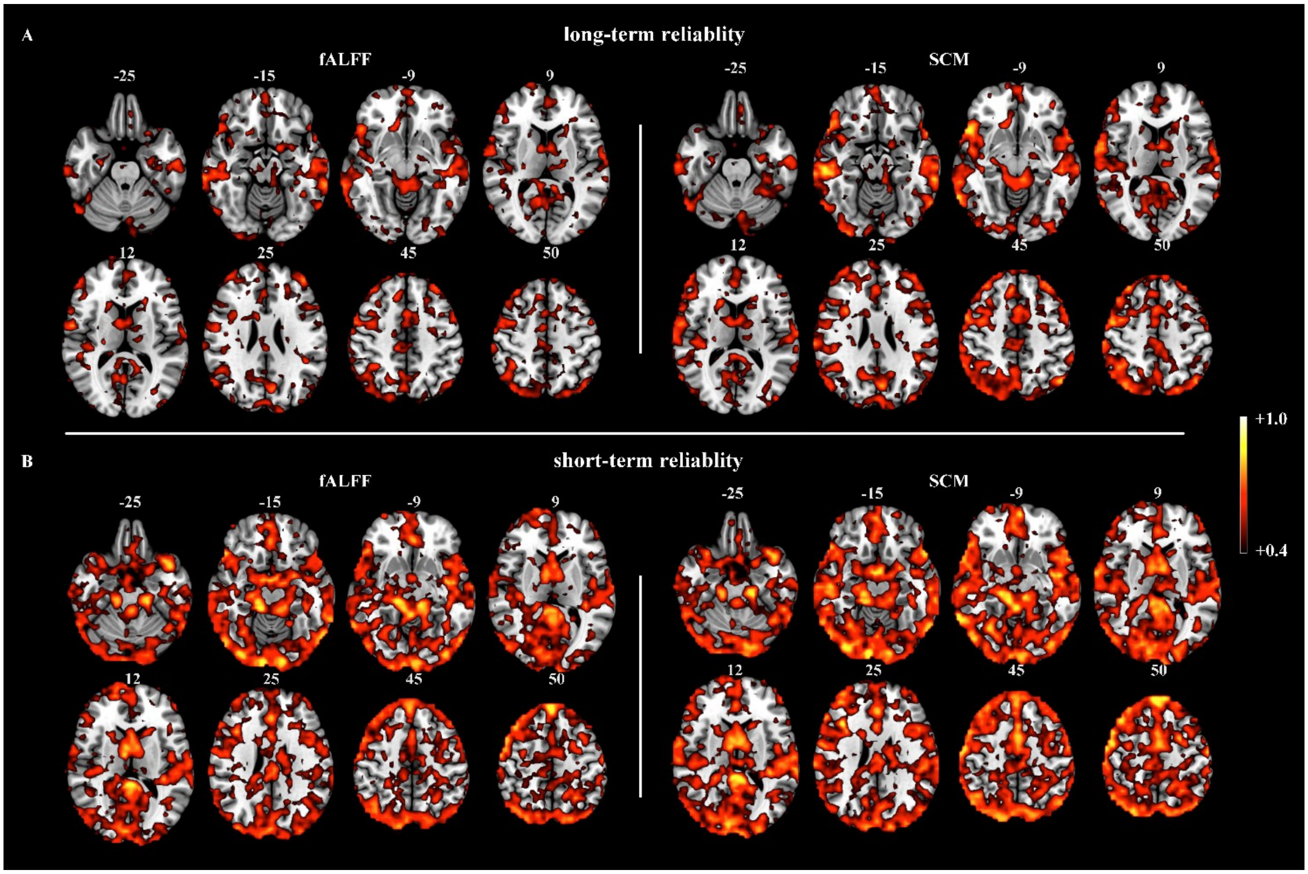

Figure S8. ICC distribution of all voxels in long- and short-term reliability with regard to fALFF and SCM on the test-retest resting-state dataset.

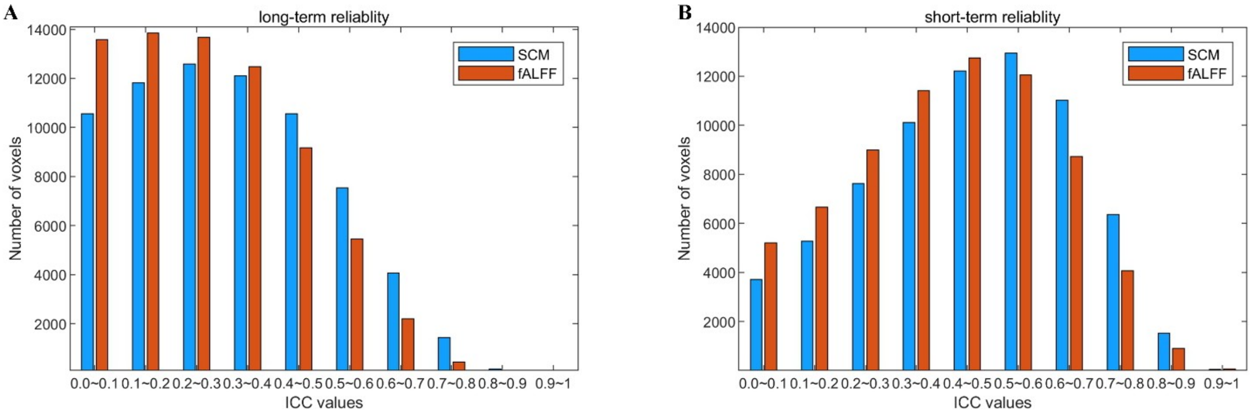

Figure S9. One-sample t-test fALFF maps under the EC (n = 45, t > 6.0, p < 0.0005, FDR corrected) and EO (n = 45, t > 6.0, p < 0.0005, FDR corrected) resting-state conditions.

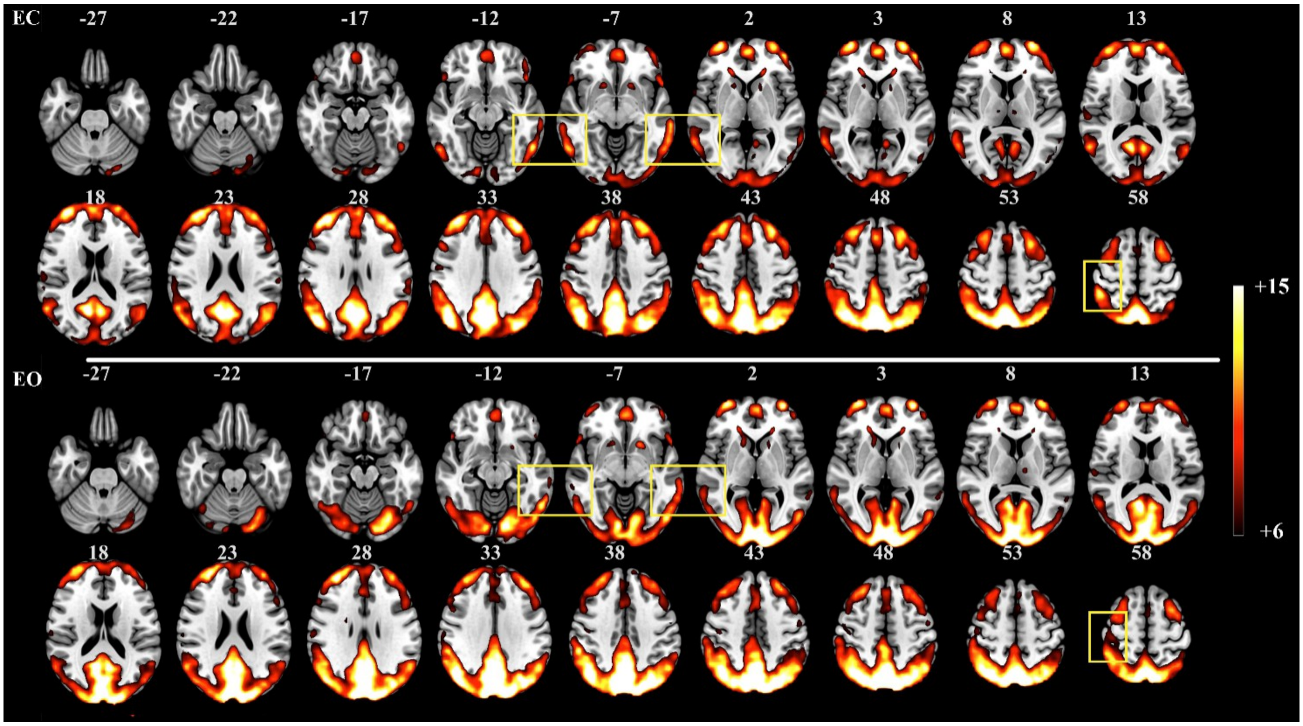

Figure S10. Paired t-test maps between SCM (hot color) and fALFF (winter color) under the EC-EO resting-state datasets without head motion regression (n = 45, t value > 6.0, p < 0.005, FDR corrected).

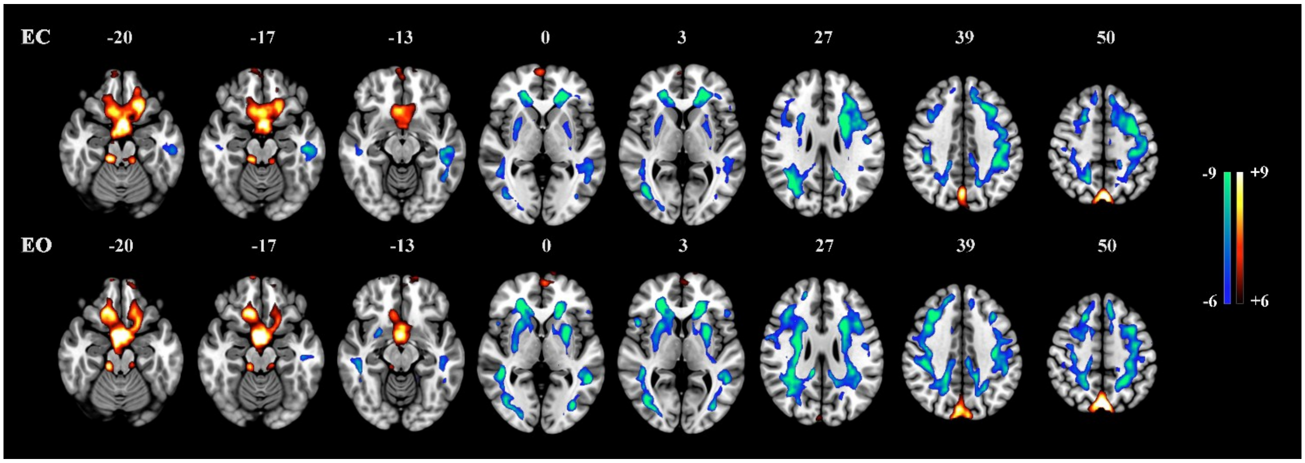

Figure S11. (A) Paired t-test maps between SCM (hot color) and ALFF (winter color) under the EC-EO resting-state datasets without head motion regression (n = 45, t value > 6.0, p < 0.005, FDR corrected). (B) Paired t-test maps between SCM (hot color) and perAF (winter color) under the EC-EO resting-state datasets without head motion regression (n = 45, t value > 6.0, p < 0.005, FDR corrected).

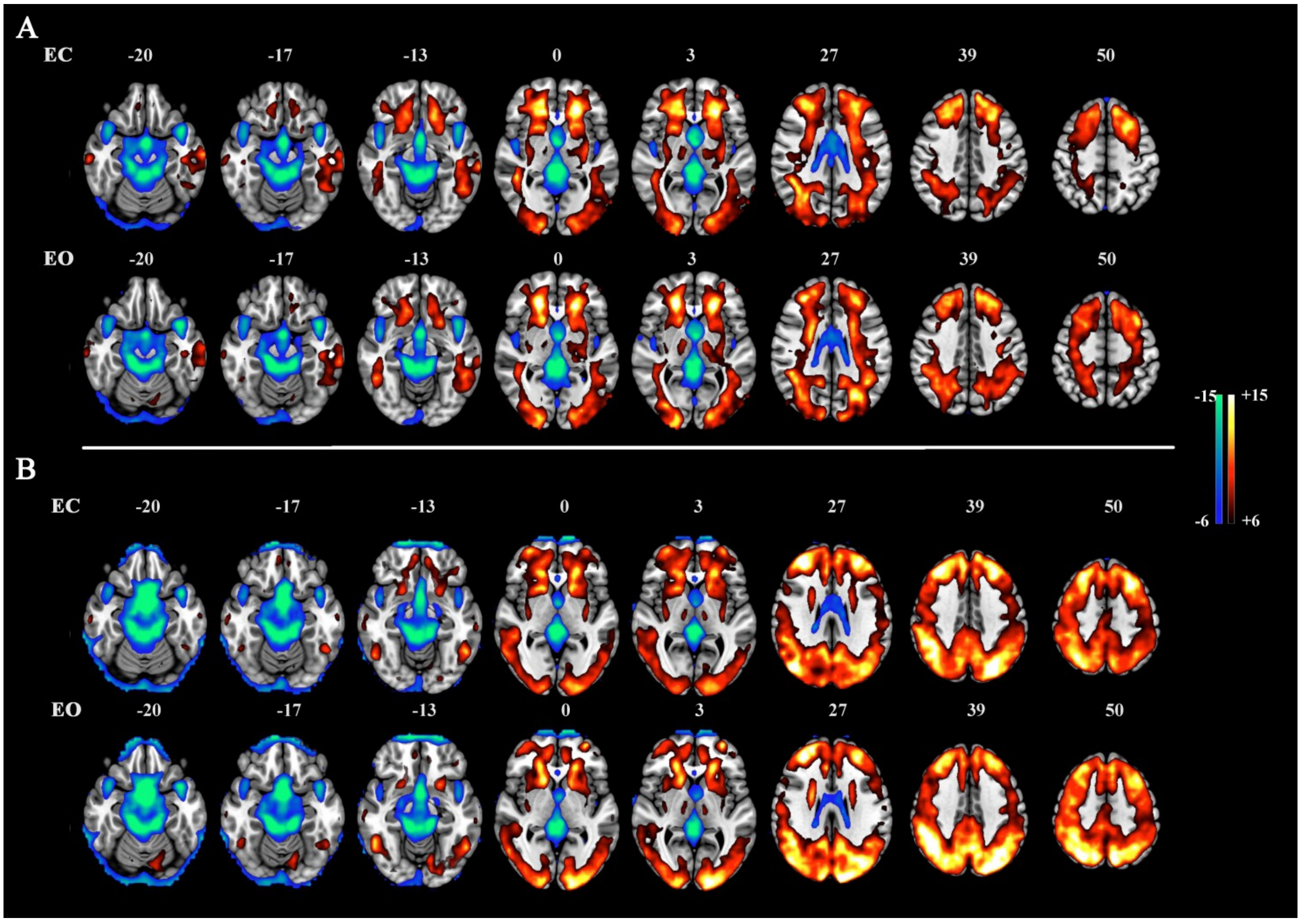
A
EC
-20
-17
-13
0
3
27
39
50
EO
-20
-17
-13
0
3
27
39
50
B
EC
-20
-17
-13
0
3
27
39
50
EO
-20
-17
-13
0
3
27
39
50
-15
+15
-6
+6
